# Flexible triboelectric nanogenerators using transparent copper nanowire electrodes: energy harvesting, sensing human activities and material recognition†

**DOI:** 10.1039/d3mh00404j

**Published:** 2023-05-15

**Authors:** Biswajoy Bagchi, Priyankan Datta, Carmen Salvadores Fernandez, Priya Gupta, Shireen Jaufuraully, Anna L. David, Dimitrios Siassakos, Adrien Desjardins, Manish K. Tiwari

**Affiliations:** a Wellcome/EPSRC Centre for Interventional and Surgical Sciences, UCL London W1W 7TS UK m.tiwari@ucl.ac.uk +442031081056; b Nanoengineered Systems Laboratory, UCL Mechanical Engineering London WC1E 7JE UK; c Elizabeth Garrett Anderson Institute for Women's Health, UCL London WC1E 6AU UK; d NIHR Biomedical Research Centre at UCL UK; e Department of Medical Physics and Biomedical Engineering, University College London Gower Street London WC1E 6BT UK

## Abstract

Triboelectric nanogenerators (TENGs) have emerged as a promising green technology to efficiently harvest otherwise wasted mechanical energy from the environment and human activities. However, cost-effective and reliably performing TENGs require rational integration of triboelectric materials, spacers, and electrodes. The present work reports for the first time the use of oxydation-resistant pure copper nanowires (CuNWs) as an electrode to develop a flexible, and inexpensive TENG through a potentially scalable approach involving vacuum filtration and lactic acid treatment. A ∼6 cm^2^ device yields a remarkable open circuit voltage (*V*_oc_) of 200 V and power density of 10.67 W m^−2^ under human finger tapping. The device is robust, flexible and noncytotoxic as assessed by stretching/bending maneuvers, corrosion tests, continuous operation for 8000 cycles, and biocompatibility tests using human fibroblast cells. The device can power 115 light emitting diodes (LEDs) and a digital calculator; sense bending and motion from the human hand; and transmit Morse code signals. The robustness, flexibility, transparency, and non-cytotoxicity of the device render it particularly promising for a wide range of energy harvesting and advanced healthcare applications, such as sensorised smart gloves for tactile sensing, material identification and safer surgical intervention.

## Abstract 2

A high performance flexible triboelectric nanogenerator using oxidation resistant pure copper nanowire electrodes.

## Graphical abstract

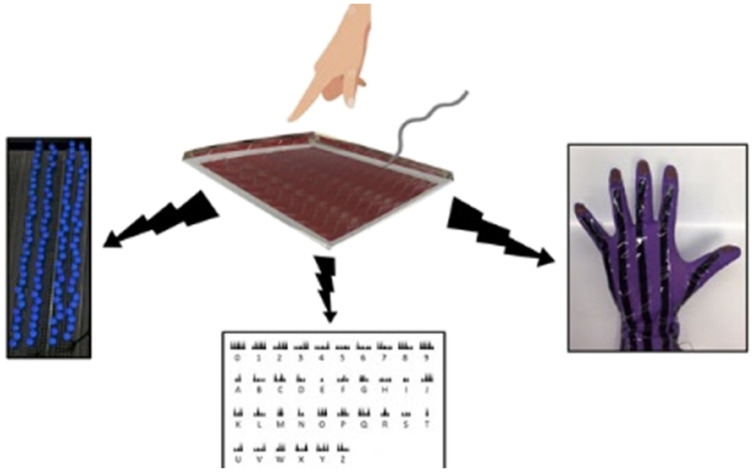

**New concepts**

Use of cheaper materials, reduced processing steps and simpler designs is essential to develop inexpensive triboelectric nanogenerators (TENGs). In this work, we have for the first time employed a simple, efficient and environment-friendly approach to synthesize and use pure copper nanowires to develop a flexible electrode for high-performance TENGs. We introduce oxygen-resistant and additive-free pure copper electrodes in the TENG, which is a major challenge for the scientific community working on TENGs. This is made possible by a combination of three simple steps: rapid synthesis of copper nanowires (15 min), vacuum filtration and lactic acid treatment, which help to maintain the high electrical conductivity and enable stable/long-term high output from the TENG after encapsulation with Ecoflex. Previous works on copper nanowire-based TENGs relied on using carbon–copper hybrids, which involves multiple complex and energy intensive steps including hydrothermal treatment, chemical vapour deposition, etching, *etc.* Our method is very simple and scalable, requiring minimal resources and technical expertise, and gives reproducible yields. The efficiency of our process is also reflected in the outstanding stability, robustness, flexibility and performance achieved relative to contemporary TENGs using a copper-based approach. Thus, the strategy described in our work offers a firsthand economic solution to exploit pure nanostructure copper electrodes in TENGs and other flexible sensor design technologies.

## Introduction

Green, sustainable and alternative power sources are being ardently sought to meet the growing demand of energy. In particular, the thriving market for wearable electronic devices and sensor networks for diverse applications ranging from energy harvesting to human health monitoring has led to novel ways of exploiting unconventional yet readily available energy sources. This requires unique strategies, both in terms of materials and technology.^1^ One such promising example is harnessing the wide assortment of ambient energy available in the form of mechanical vibrations, fluid motion and human activities. Therefore, energy harvesters and sensors based on electromechanical transformative technologies, such as triboelectric nanogenerators (TENGs) and piezoelectric nanogenerators (PENGs), are being explored to convert the ubiquitous mechanical energy from the natural environment into electrical power.^1,2^ Among these, TENGs relying on the coupling effect of contact electrification and electrostatic induction have been demonstrated to be economically viable and energy-efficient because of their innate advantages of simple fabrication, wider choice of materials, simple operation, high efficiency and large output power.^1–3^

Over recent years, a number of fabrication processes have been investigated to improve the performance of TENGs, by introducing various modifications and desirable attributes in terms of rational choice of materials, light weight, mechanical robustness, flexible electrodes and device design.^4^ However, achieving optimum and stable electrical output over a long period of time still remains a challenge due to various factors such as degradation of the triboelectric layer under repeated contact and separation, and poor electrical contact between the electrodes and the triboelectric layer.^4^ One of the recent developments in TENG design is the use of flexible electrodes working in the so-called single electrode configuration, where one triboelectric layer (stationary layer) is attached to the electrode and the movable counter layer is free and serves as the grounding electrode.^5^ This substantially reduces the device design complexity, which is particularly welcome when it comes to tactile sensors that not only detect body motion but can also be used to harvest mechanical energy directly from the human body, specifically when in direct contact with skin, a well-known triboelectrically positive material.^5^ Thus, combining an appropriate triboelectrically negative layer with a flexible electrode presents a promising way of developing self-powered smart tactile sensor pads and wearable energy harvesting devices. However, the performance of such single electrode based TENGs is critically dependent on the nature, conductivity, contact, flexibility and compatibility of the electrode with the stationary triboelectric layer.^4,5^ For example, Shankaregowda *et al.*^6^ used graphite-coated paper as the electrode material for polydimethylsiloxane (PDMS)-based single electrode flexible TENGs and were able to generate an open circuit voltage of 320 V. In a separate study, conducting polypyrrole was coated on cotton textile to make a single electrode textile-based TENG.^7^ However, difficulty in processing exacerbated by poor mechanical stability and electrical conductivity severely limits the long-term use of such carbon or conductive polymer-based electrodes in TENGs.^8^ Thus, to address these issues of mechanical strength, flexibility and high electrical conductivity at the microstructure level, metallic nanowire-based electrodes have been explored. For instance, flexible TENGs were fabricated with silver nanowires sandwiched between PDMS, which operated under the single electrode mode configuration.^9^ Silver nanowires were also embedded in PDMS and thermoplastic polyurethane to obtain self-healing TENGs.^10^ However, more recently, nanostructured copper-based electrodes have been gaining attention as an abundant and cheaper alternative to silver. Copper nanowires (CuNWs) have an electrical conductivity that closely competes with that of silver (94.6% of Ag), but with obvious economic benefits: copper is 1000 times more abundant and its cost is <10% that of silver.^11^ In the field of flexible electronics, copper nanoparticle/nanowire based electrodes have been widely used to form excellent electrical interconnects, while also offering mechanical flexibility and transparency.^11,12^ However, a persistent drawback is that copper in nano sizes is highly reactive and oxidises under ambient conditions, which drastically reduces their electrical conductivity and hence long-term stability.^13^ Incidentally, Zhang *et al.*^14^ prepared electrodes for TENGs by evaporating bulk copper (thickness 500 nm) on sandpaper to enhance friction and power output. More recently, flexible and transparent TENGs were fabricated by using single crystal graphene^15^ and reduced graphene oxide (rGO)^16^ coated copper nanowires as flexible electrodes. An important point to note is that despite remarkable properties, CuNWs almost always require protective coatings in the form of metals, conducting polymers, oxides, amorphous carbon, *etc.*, to prevent oxidation.^17–20^ This not only poses a challenge by reducing electrical conductivity, but can also lead to undesirable contributions from additional materials and post processing steps during TENG fabrication.^16^ Although pure Cu nanowire–Cu mesh composite electrodes have been prepared for PDMS-based TENGs, they were not flexible and required multiple energy intensive processing steps.^21,22^

**Fig. 1:**
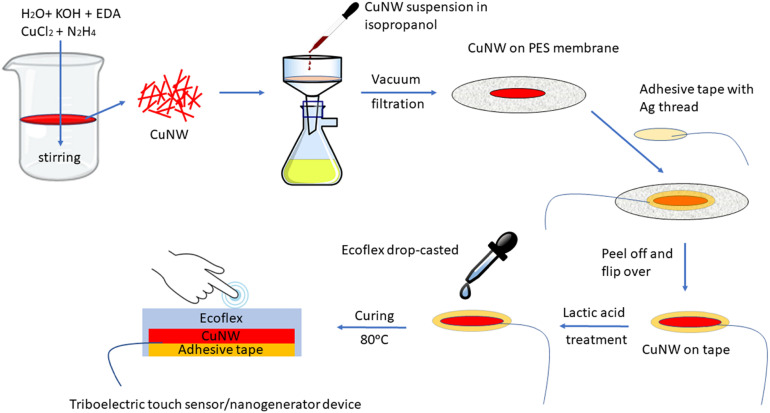
Schematic for the fabrication of the CuNW-Ecoflex based single electrode TENG.

As a solution to these major problems, we fabricated a flexible and transparent TENG by directly introducing a conducting layer of pure copper nanowires (CuNWs) as a flexible electrode without using any protective agent or polymers at room temperature. This simple process, schematically shown in Fig. 1, involves vacuum filtration and transfer of the as synthesized bare CuNW layer on an adhesive tape (40 μm thickness) followed by washing with lactic acid and encapsulation inside an elastomer, Ecoflex 00-50 (serving as the triboelectric layer). This procedure effectively prevents the oxidation of CuNWs and preserves its conducting properties without involving any additives. The whole synthesis and TENG fabrication process does not involve any time consuming and energy intensive steps like hydrothermal treatment, spin and spray coating, chemical vapour deposition, and etching as commonly observed in recent reports. To the best of our knowledge, this is the first report of using pure chemically synthesized oxidation resistant pure copper nanowires as a flexible and transparent electrode for TENGs using a simple and scalable strategy requiring minimal resources and technical expertise. Our lactic acid based approach is also environmentally friendly as it is widely used in the food industry and is easily metabolised by humans and animals.^23^

## Results and discussion

**Fig. 2:**
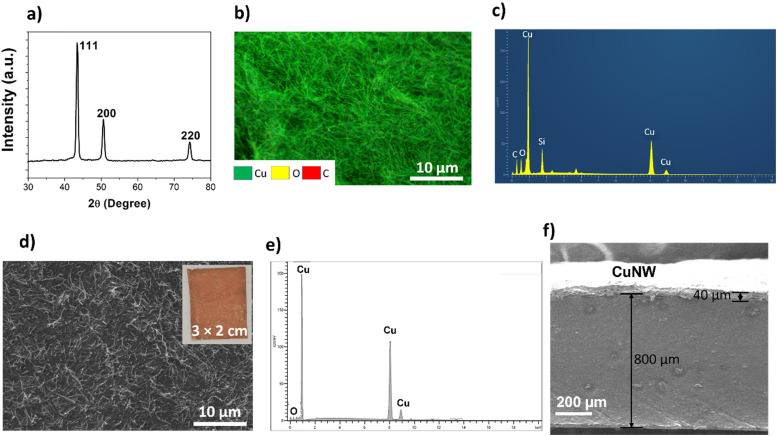
(a) XRD pattern of the CuNWs, (b) SEM and elemental mapping of the as synthesized CuNWs, (c) EDS spectra of the CuNWs before lactic acid treatment, (d) SEM of the CuNW electrode layer after lactic acid treatment (inset-image of the electrode on adhesive tape), (e) corresponding EDS spectra and (f) cross-section SEM image of the TENG showing the Ecoflex and CuNW layer.

To realize the flexible CuNW electrode, a bottom-up chemical approach was employed to synthesize the CuNW by using KOH and ethylenediamine as the growth promoter and hydrazine hydrate as the reducing agent respectively. The ratio of KOH/ethylenediamine plays a crucial role in determining the aspect ratio of the wires. In the present case, a high ratio is selected for optimum nanowire growth.^24^ Interestingly, by replacing sodium hydroxide as previously reported^24^ with KOH, the reaction time can be significantly reduced to 15 min. This is because the low viscosity of the KOH solution leads to faster interaction between the copper salt and the reducing agent resulting in expedited formation of copper nanowires. It should be noted that the synthesis route requires high concentration of corrosive alkali (KOH) solution and hence proper safety measures should be observed during synthesis. Fig. 2(a) shows the XRD pattern of the as synthesized copper nanowires with peaks at 43.2°, 50.3° and 74.1°, respectively. This corresponds to a face centered cubic (fcc) structure (JCPDS card no 03-1005). Additionally, there is no detectable peak of any other phases such as oxides or hydroxides. Scanning electron microscopy (SEM) images of the as synthesized CuNWs show densely packed nanowire morphology, with average wire diameter 60 nm and length in the 50–100 μm range (Fig. 2(b)). Elemental mapping of the same area (Fig. 2(b)) shows mainly copper with some oxygen phases. However, the corresponding EDS spectrum (Fig. 2(c)) shows a considerable proportion of oxygen phase. This indicates that a certain amount surface oxidation or hydroxide formation has occurred on the as synthesized CuNWs as no protective agent was used.

The main limitation of copper nanowire-based electrodes is their high propensity towards oxidation in air, which drastically reduces the electrical conductivity. Generally, bare copper nanowires are rapidly (within minutes) covered with a thin layer of oxide or hydroxide upon contact with air. Removal of this layer can help recover the electrical conductivity. In our preparation of the CuNW electrode for TENGs, the as synthesized copper nanowire was simply washed with a dilute solution of lactic acid to expose the underlying metallic copper. As observed by Won *et al.*,^25^ lactic acid initially reacts with the outer copper oxide or hydroxide layer to form copper lactate, which can then be easily washed away by isopropanol to expose the underlying copper. This procedure, which takes only a minute, does not require any post processing such as thermal annealing or reduction under Ar or N_2_ atmosphere. The washed copper is stable for 2 h at room temperature and shows very low sheet resistance (6 Ω cm^−2^) compared to the untreated CuNW layer (1 MΩ cm^−2^). Fig. 2(d) shows the SEM image of the CuNW electrode layer on adhesive tape after treatment with 1% lactic acid (inset – image of a ∼6 cm^2^ CuNW electrode layer on tape). The morphology is maintained except that the nanowires are shorter in length (5–15 μm) due to the ultrasonic treatment prior to vacuum filtration, which breaks the wires down. In contrast to the as synthesized CuNWs, the EDS spectra (Fig. 2(e)) of the lactic acid-treated CuNW layer show predominant peaks of copper with significantly reduced oxygen content.

Following lactic acid treatment, rapid encapsulation with Ecoflex and curing in a vacuum oven removes the possibility of exposure to air, thus providing chemical stability to the CuNW electrode layer. A cross-section view of the TENG shows an approximately ∼40 μm thick CuNW layer encapsulated by Ecoflex (∼800 μm) (Fig. 2(f)).

**Fig. 3:**
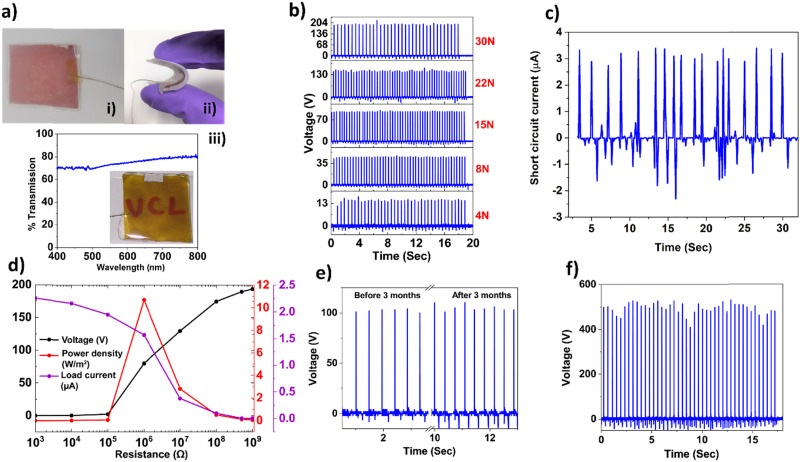
(a) Photograph of the TENG showing (i) a 6 cm^2^ device, (ii) flexibility and transparency on (iii) normal adhesive tape, (b) *V*_oc_ obtained under different finger tapping forces up to 30 N, (c) maximum *I*_sc_ obtained under a tapping force of 30 N, (d) variation of the voltage, current and power density under different load resistances, (e) stability of the TENG before and after 3 months and (f) maximum *V*_oc_ obtained from a 5 cm × 5 cm TENG under a finger tapping force of 30 N.

Fig. 3(a)-(i) shows a single electrode TENG fabricated by sandwiching a layer of CuNW between Ecoflex and adhesive tape as already described above. The red coloration of the device is due to the layer of CuNW under the Ecoflex. The TENG is very light weight (1.12 g) and flexible (Fig. 3(a)-(ii)) with an active area of ∼6 cm^2^, which can be easily made transparent by using a lower concentration of copper nanowires on Kapton adhesive tape as the flexible substrate. As evident from Fig. 3(a)-(iii), the transparent TENG shows *a*% transmission of around 75% in the UV-visible spectrophotometer. (Inset: Transparent TENG placed on top of the letters ‘UCL’).

To evaluate the performance of our device, we measured the open circuit voltage (*V*_oc_) under different finger tapping force (4 N–30 N) using the non-transparent TENG. As evident, the *V*_oc_ increased with the tapping force and the flexible TENG produced a maximum *V*_oc_ of 200 V (Fig. 3(b)) and a corresponding short circuit current (*I*_sc_) of 3.4 μA (Fig. 3(c)). The output performance of the device was further investigated with different load resistances (10^3^–10^9^ Ω). Under the same tapping conditions (30 N, 2 Hz), the instantaneous voltage increased and gradually reached a peak value of 194 V at 10^9^ Ω, while the corresponding current measured across load resistance *R*_L_ decreased (Fig. 3(d)). Effective power density across a pure resistive load is determined from the equation 
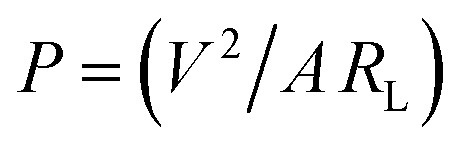
, where *A* is the effective surface area (width × length).^26,27^ The device gave a maximum peak power density of 10.67 W m^−2^ with 1 MΩ load (Fig. 3(d)). In terms of output voltage and power density, this is the highest reported value achieved from a device of size around 6 cm^2^ that uses a pure copper nanowire-based flexible electrode. Table S1 (ESI†) summarizes the performance of metal nanowire-elastomer (polydimethylsiloxane (PDMS))-based TENGs from the literature with respective parameters like maximum output voltage (*V*_oc_) and current (*I*_sc_), area of the device and power density as compared to our work. We believe that the superlative performance of our TENG is a direct consequence of using the pure copper nanowire and Ecoflex combination, which makes multiple positive contributions. First, the unoxidized surface of the copper nanowire provides better electrical conductivity compared to the hybrid electrodes, where copper is coated/mixed with graphene or conducting polymers, thus serving as an efficient charge generating and collecting layer when in contact with triboelectric Ecoflex.^4^ Second, due to the superior charge trapping capability of Ecoflex (because of physical and chemical traps in the polymer structure), it effectively suppresses ‘charge decay’ by impeding the neutralization rate of the negative triboelectric charge and induced positive charge on the CuNW electrode. Third, as Ecoflex is softer, more stretchable and flexible than PDMS, it can undergo large interfacial deformation, which increases the area of contact and enhances the charge density during finger tapping.^27^

Under similar tapping conditions, the transparent TENG produced a maximum *V*_oc_ of 125 V (Fig. S1a, ESI†) and corresponding *I*_sc_ of 1.1 μA (Fig. S1b, ESI†) and a maximum power density of 3.38 W m^−2^ (Fig. S1c, ESI†) at 1 MΩ. Although the performance of the transparent TENG is lower, it is comparatively better than for previously reported TENGs (see Table S1, ESI†). This is expected, as in the transparent TENG, with significantly less CuNWs in the electrode layer, the density of induced positive charges on the electrode will be lower and hence the overall power density is lower.^4^ Thus, controlling the concentration of CuNW in the electrode layer provides a way to realize an optimum trade-off between transparency and electrical power generation tailored for specific applications.

**Fig. 4:**
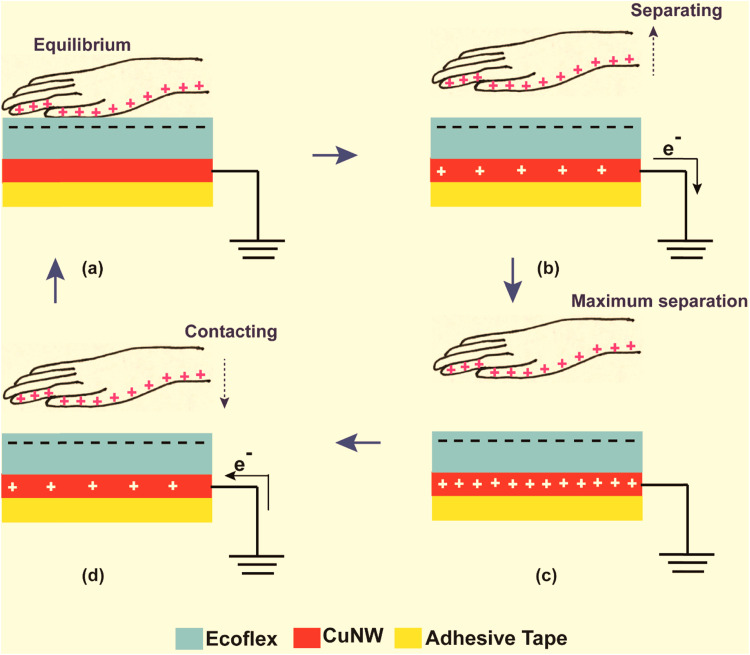
Schematic showing the working principle of the CuNW-Ecoflex TENG.

The working principle of the as fabricated single electrode TENG is schematically represented in Fig. 4(a) and (d). Energy harvesting from mechanical tapping occurs through a coupled effect of contact electrification and electrostatic induction between human skin and a triboelectrically negative layer (in this case Ecoflex).^28,29^ The consecutive steps of electric power generation process in the TENG can be briefly described as follows. Initially, the surface of Ecoflex is in full contact with human skin leading to charge transfer between them.^5,30^ According to the triboelectric series,^31^ Ecoflex gets negatively charged while the human skin surface is positively charged due to the tribo-electrification phenomenon. However, there is no electron flow through the external circuit due to balancing of opposite charges (Fig. 4(a)). Once separation of Ecoflex and human skin occurs, these triboelectric charges are not compensated, and hence the negative charges on the surface of Ecoflex induce positive charges on the CuNW electrode, resulting in flow of free electrons from the electrode to the ground (Fig. 4(b)). This electrostatic induction step produces an output voltage/current signal, which becomes zero when negative triboelectric charges on Ecoflex almost nullify the induced positive charges on the CuNW electrode as the separation increases (Fig. 4(c)). When the human skin again approaches Ecoflex, the induced positive charges on the CuNW electrode decrease and the electrons flow from the ground to the CuNW electrode until the human skin and Ecoflex are fully in contact (Fig. 4(d)). This results in the reversed output voltage/current signal.^5^

**Fig. 5:**
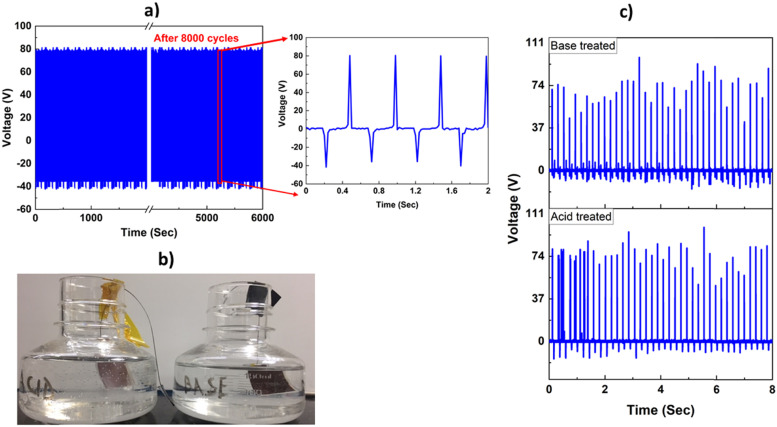
(a) Stability test of the TENG under continuous operation for 8000 cycles in single electrode mode (frequency: 4 Hz, force: 30 N). PES membrane was used as the counter triboelectric surface, (b) chemical stability test set-up for the TENG using 10 (M) sulphuric acid (left image) and sodium hydroxide (right image) and (c) *V*_oc_ measured after 72 h of acid and base submersion.

As already mentioned, copper electrode-based TENGs to date have relied on copper sourced from bulk form (such as copper foil tape or metal evaporation) or in combination with different coating agents in the case of nano sized copper. The coatings, however, affect conductivity.^14–16,20^ In our TENG, once the Ecoflex layer is cured on the lactic acid treated CuNW electrode, the device has long term stability in ambient atmosphere, which is evident from the stable *V*_oc_ observed with the same device before and after 3 months of room condition storage (tested with 15 N finger tapping force at 4 Hz, see Fig. 3(e)). Furthermore, the easy fabrication of the device is an obvious advantage with respect to scalability as can be seen from Fig. 3(f), where we fabricated a 5 cm × 5 cm device (using the same method described in Fig. 1, which gave a maximum *V*_oc_ of 523 V under a tapping force of 30 N. The device can be made as large as possible depending on the diameter of the commercially available Buchner funnel and filter membrane. Our TENG is inherently flexible due to the Ecoflex-CuNW-adhesive tape layered structure, all of which are pliable, and the adhesive tape can be replaced with any other flexible substrate (of different shape and sizes) of choice such as black insulating tape, Gorilla tape, masking tape, double sided tape and Kapton tape depending on the application (Fig. S2, ESI†). To assess stability, the performance of the device was further tested by repeated impact using a pneumatic linear actuator fitted with a PES membrane (as the counter triboelectric surface) and controlled by Arduino (Video S1, ESI†). Under a force of 30 N and frequency of 4 Hz, the TENG maintained an average *V*_oc_ of 80 V for 8000 cycles in single electrode mode (Fig. 5(a)). A lower value of *V*_oc_ is obtained as the PES membrane is less triboelectrically positive than human skin. We also tested the robustness and flexibility of the device (area: 7 cm × 1 cm) by repeated stretching (at 4 Hz frequency, for 5000 cycles) using a motorized stage and bending and twisting by hand. Thereafter, a stable *V*_oc_ of 80 V obtained under finger tapping clearly indicates the robustness of the device (Video S2, ESI†). Furthermore, to evaluate the stability of the TENG under harsh chemical exposure, we submerged the TENG in 10(M) solution of sulphuric acid and sodium hydroxide solution (Fig. 5(b)) at 65 °C for 96 h under ambient conditions, followed by washing and drying.^32^ Both the treated TENGs produced an average *V*_oc_ of 75 V under a finger tapping force of 10 N (Fig. 5(c)). This also shows that in our design, the Ecoflex layer completely seals the CuNW electrode, protecting it from the external environment. The device is also very versatile: it produced a stable *V*_oc_ when contacted with different triboelectrically positive materials like PES (116 V), nitrile rubber (106 V) and Nylon (164 V) under single electrode mode configuration (Fig. S3, ESI†). This has implications to develop cost effective TENGs and sensors by combining our device with triboelectric polymers commonly used in packaging and healthcare industries.

**Fig. 6:**
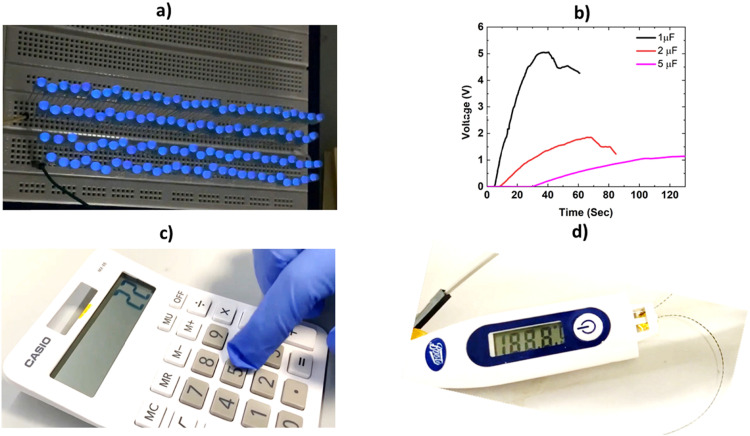
TENG applications – (a) powering up 115 LEDs, (b) charging capacitors, (c) powering a digital calculator and (d) a thermometer.

The developed TENG was then used to explore various practical applications to substantiate its utility. The TENG could generate sufficient power to light up 115 blue LEDs under human finger tapping (15 N, 2 Hz) (Fig. 6(a) and Video S3, ESI†). Under the same conditions, the transparent TENG is able to power up 50 LEDs (Video S4, ESI†). The device is also able to charge 1 μF, 2.2 μF and 5 μF capacitors within a very short amount of time (Fig. 6(b)) which is utilized to power up the LED screen of a digital calculator (Fig. 6(c)) and thermometer (Fig. 6(d)) (Video S5 and S6, ESI†).

The TENG was also exploited as a self-powered Morse code signal generator using finger tapping. Morse code is a simplistic yet widely used communication method for emergency communication through radio waves.^33^ Morse code makes use of dots and dashes to form different letters. We employed our TENG for possible applications in emergency and surveillance scenarios, by using the voltage signals created by manually tapping on the TENG in two distinct manners: either a light touch or a harder tap. The lighter touch would correspond to a dot in Morse code while the harder tap would correspond to a line. The voltage signals created by the two distinct tapping modes as measured from the oscilloscope were then fed into our signal analysing algorithm developed using Python language in PyCharm (Fig. S4, ESI†), which takes into account certain features based on the amplitude of the voltage peaks and classifies them to obtain dots and dashes and the corresponding letters they represent in Morse code to encode words like “WEISS” which stands for Wellcome/EPSRC Centre for Interventional and Surgical Sciences (Video S7, ESI†).

Wearable force and motion sensing devices have been gaining tremendous interest, especially in the biomedical field for monitoring human health.^2,34,35^ Interestingly, our TENG, when applied on the skin can detect stretching motion (Video S8, ESI†) when the fist is repeatedly opened and closed. Similarly, it can also detect the bending motion of the wrist and finger joints (Videos S9 and S10, ESI†). The sensing mechanism is similar to that described in the context of Fig. 4, whereby stretching and bending cause changes in frictional contact between the skin and the sensor resulting in a voltage signal. Thus, the flexibility, triboelectric performance and sensing capability of the device offer opportunities to use them as self-powered devices to not only harvest energy from human body motion but also to detect muscle flexing, joint movements *etc.*, which is needed for intelligent robots and rehabilitation monitoring applications.

**Fig. 7:**
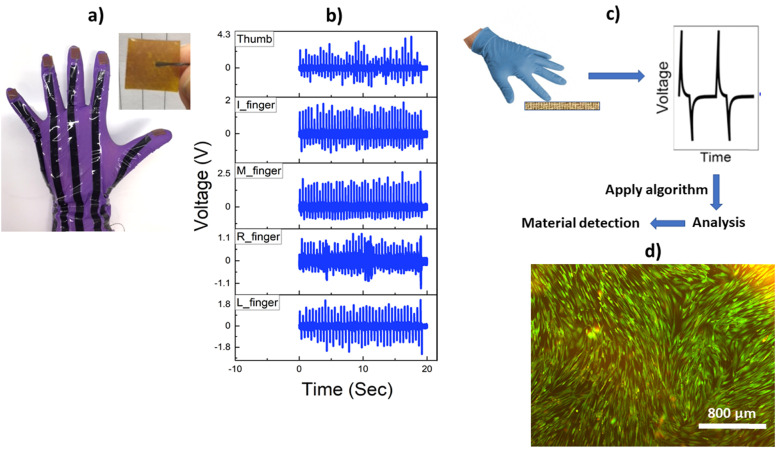
(a) Image of the TENG sensorized surgical glove with flexible interconnects, (b) *V*_oc_ obtained from tapping with the glove on skin, (c) material identification with the TENG mounted glove and (d) fluorescence microscopic image of live human dermal fibroblasts (HDFs) after treatment with the TENG for 48 h, showing excellent biocompatibility.

To further highlight this, as a proof-of-concept, we developed a glove (using silver coated threads as flexible interconnects) integrated with one of our flexible TENGs (size: 1 cm × 1 cm) at each finger-tip (Fig. 7(a)) to record the voltage output (Fig. 7(b)), which directly corresponds to the force applied by each finger upon tapping on skin. As can be seen from Fig. 7(b), tapping with the little, middle and index finger generated consistent peaks of equal intensity while, the ring finger and thumb produced peaks of non-uniform intensities, which indicates that some fingers are dominant and the different orientation of the thumb with respect to the other fingers might be responsible for this. The result will of course vary from subject to subject and might provide useful information about how a human hand interacts with objects. This tactile sensing ability of the glove was also explored by grasping different objects (*e.g.*, marker pen, scissors, glass vial, plastic tube, scalpel, torch) with the glove and the *V*_oc_ was recorded corresponding to a specific finger involved in the interaction. When each object was repeatedly grasped by a specific finger (here the index finger was tapped while other fingers were holding the object), they produced different *V*_oc_ values due to triboelectrification between Ecoflex and the surface of the object, potentially highlighting object recognition capability through tactile feedback (Fig. S5, ESI†). Furthermore, variation of *V*_oc_ was recorded by gently tapping on different surfaces simultaneously with four fingers (excluding the thumb). As can be seen from the video (Video S11, ESI†), when tapping was continued from silicone surface to plywood, different patterns were observed. *V*_oc_ was found to depend on two factors – type of finger used and the surface contacted. Under the same tapping force, the ring finger (cyan line in DSO, Video S11, ESI†) produced the highest signal followed by the middle finger (purple line in DSO, Video S11, ESI†), index finger (green line in DSO, Video S11, ESI†) and little finger (yellow line in DSO, Video S11, ESI†) irrespective of the surface contacted. And in terms of surfaces, tapping on silicone generated the highest signal while the least output was recorded on plywood (Video S11, ESI†). Although further investigation is required in the future, these preliminary studies show that the TENG integrated (sensorised) glove has a strong potential to be used as a training tool to recognize objects through tactile feedback and possibly aid clinicians to estimate the force applied on medical devices and organs during surgical procedures.^36^ Furthermore, this could also help acquire haptic and tactile feedback related to medical conditions such as Parkinson's disease, which currently use bulky electronic and magnetic coil-based sensors.^37^ As can be seen from the voltage vs force curve (Fig. S6, ESI†) our TENG is capable of responding to a wide range of forces (4–30 N), which is suitable for the above stated applications. Exploiting the tactile sensing capability of the TENG, we were also able to detect and identify materials by using a simple algorithm developed in MATLAB. We first selected different materials and recorded voltage and polarity (determined by the direction of the first peak upon contact with the TENG – upward and downward peak indicates ‘+’ and ‘−’ polarity, respectively). The data is then compiled to form a material database. In the second step, voltage and polarity are recorded for a known sample. We then use an algorithm, which compares each material with the sample based on voltage and polarity differences. The result is represented in Table S2 (ESI†) which shows that the sample has the least difference (0.2 V and same polarity) with Kapton film. The sample is indeed a polyimide film (Goodfellow, UK) which confirms that it is identical to Kapton (a brand name for polyimide). The glove was able to detect polyimide with a success rate of 98% after the experiment was repeated 20 times. As also reported by Wang *et al.*,^38^ tactile sensing-based material identification is an emerging field that could be applied in the simplest way to recognize material properties without using sophisticated analytical instruments. Although rigorous studies need to be undertaken to understand specificity, sensitivity, surface area, force, temperature and moisture effects, our glove-based approach with a finger mounted thin flexible TENG holds promise as a user-friendly sensing tool in broader materials/engineering applications.

Before realizing the above-mentioned potential of our TENG in advanced biomedical sensing and flexible electronic skin type applications, which involve direct human contact with the device, it is essential to ensure biocompatibility with tissues or cells. While silicone-based elastomers like PDMS and Ecoflex are widely recognized as biocompatible,^39^ metallic electrodes especially containing nanostructured copper or silver may exhibit cytotoxicity at higher concentrations when exposed to biological tissues due to their high reactivity.^40^ To evaluate this, the biocompatibility of the developed TENG was assessed on the human dermal fibroblast (HDF) cell line by directly incubating the device with an adherent culture of cells for 72 h. The viability is determined from the proportion of green (live) and red (dead) cells, which have been dual stained with Calcein AM and ethidium homodimer, respectively.^41^ As evident from Fig. 6(d), predominantly green cells with elongated morphology, characteristic of healthy growing fibroblast cells, are observed indicating that the TENG is noncytotoxic as there is no significant leakage/leaching from the CuNWs. Thus, the Ecoflex serves as an affective protective layer for the CuNW electrode making it potentially suitable for future *in vivo* applications.

## Conclusions

A flexible, energy-efficient and easily scalable TENG has been developed using low-cost copper nanowires as a potent electrode material and commercial Ecoflex as the triboelectric layer. A new strategy involving vacuum filtration and lactic acid treatment is employed to obtain oxidation resistant pure copper nanowires with excellent electrical conductivity. This enabled us to make a single electrode TENG yielding *V*_oc_ ∼ 200 V and a power density as high as 10.67 W m^−2^, using an ∼6 cm^2^ device, far superior to reported copper nanocomposite based electrodes. This is further attributed to the enhanced charge trapping and collection efficacy of the Ecoflex-CuNW combination. The CuNW electrode layer can be transferred on any adhesive substrate and made transparent by using dilute suspensions, thus making it versatile for diverse applications. The elastomeric Ecoflex-CuNW layer is robust and is demonstrated to withstand mechanical stress for thousands of cycles and strongly corrosive (pH) environments. The single electrode design of the TENG and the triboelectrification mechanism enable direct energy harvesting from human finger tapping, powering >100 LEDs and electronic devices like a digital calculator and thermometer. The TENG is also utilised to generate, send and convert Morse code signals into short meaningful words by simply tapping on the Ecoflex layer. Furthermore, the TENG also shows reliable tactile sensing capabilities from human hand bending and stretching motions. This was demonstrated by developing a smart glove with integrated TENGS on each finger-tip to monitor human touch derived force/pressure as well as material identification. Future applications may involve monitoring tactile forces during surgical procedures, gesture detection, motor neuronal disease rehabilitation assessment, defence and security. The TENG showed negligible cytotoxicity towards human dermal fibroblasts, which should alleviate concerns regarding biocompatibility. Thus, with a versatile set of functions and robustness, the present CuNW-based TENG offers promise for sustainable economic production of smart devices in human/computer interfaces, skin-integrated electronics, wearable/patchable self-powered sensory systems and passive energy harvesting modules for future healthcare applications.

## Experimental section

### Materials

Copper(ii) chloride (CuCl_2_) (anhydrous, powder, ≥99.995% trace metals basis, Sigma Aldrich), Ethylenediamine (EDA) (C_2_H_8_N_2_) (≥99%, Sigma Aldrich), Hydrazine hydrate (N_2_H_4_) (reagent grade, 50–60%, Sigma Aldrich), Potassium hydroxide (KOH) (reagent grade, 90%, flakes, Sigma Aldrich), l-(+)-lactic acid solution (Sigma Aldrich), Ecoflex 00-50 (Smooth-On Inc.), Dulbecco's Modified Eagle Medium (DMEM), Phosphate buffered saline (PBS), Ethylene diamine tetraacetic acid (EDTA), Trypsin, Fetal bovine serum (FBS), antibiotic mixture (PSN) (Sigma Aldrich), and a live/dead assay kit (L3224, Thermofisher Scientific) were used.

### Synthesis of copper nanowires

Synthesis of copper nanowires (CuNWs) was carried out as described earlier with a slight modification.^21^ Briefly, KOH (64.32 g) was dissolved in distilled water (80 mL) in a jacketed glass beaker under constant stirring at 85 °C. Next, EDA (400 μL) was added followed by CuCl_2_ (4 mL, 0.1(M)). After brief mixing, hydrazine hydrate (110 μL) was added and the whole solution was stirred until the bubbles disappeared. The mixture was then left at room temperature without stirring. The solution gradually turned from transparent to deep red and after 15 min, the copper nanowires accumulated as a layer on top of the mixture (Fig. 1). The nanowires were then collected after centrifuging three times with water and finally with isopropanol. They were then stored in hydrazine hydrate (3% v/v) solution for subsequent use.

### Characterization

The X-ray diffraction (XRD) pattern of the as synthesized CuNWs was recorded by Rigaku, MiniFlex 600 using CuKα radiation (1.5409 Å) and a scan range (2*θ*) from 0 to 80° (at 40 kV). The CuNWs were vacuum dried at 40 °C for 1 h and directly used for XRD. Transmission characteristics were measured using a UV-visible spectrophotometer (Orion AquaMate, 8100). Morphological characteristics of the CuNWs were observed by a scanning electron microscope (SEM) (Zeiss, EVO 25). A small portion of the dried CuNW was directly placed on a carbon coated grid and then sputter coated with gold and observed at 20 kV. Additionally, SEM of the CuNW electrode was followed by energy dispersive spectroscopy (EDS) and elemental mapping to verify elemental composition.

### Fabrication of the TENG

The device was fabricated by using commercial elastomer Ecoflex 00-50 as the triboelectrically negative layer with human skin serving as the tribo-positive counterpart. Initially, a CuNW suspension (0.5% w/v) was prepared by ultrasonicating (100 W, 30 KHz) in 5 mL isopropanol for 2 min (5 s on and 3 s off cycle). Thereafter, 1 mL of the suspension was subject to vacuum filtration using a polyether sulphone (PES) filter membrane connected to a vacuum pump (through a Buchner funnel). Next, the layer of CuNWs was transferred onto a double sided adhesive tape (Banner Ltd) (active area: 3 cm × 2 cm) by uniformly pressing the tape and peeling it off, thus forming the electrode. The electrode layer was then soaked with a 1% solution of lactic acid for 1 min. A highly conducting layer of CuNWs was obtained after repeated washing with isopropanol and drying at room temperature. Next, a conducting silver thread was attached onto the electrode and 3 g of Ecoflex 00-50 was quickly drop-cast to cover the electrode placed on a Teflon sheet. Finally, the flexible TENG was obtained after curing the elastomer at 60 °C for 1 h in a vacuum oven (any excess Ecoflex was trimmed off from the sides). To prepare the transparent TENG, a similar procedure was followed using 1 mL of a 10-fold diluted suspension of CuNW on Kapton tape. The fabrication of the device is presented schematically in Fig. 1.

### Fabrication of the glove

Five individual TENGs (1 cm^2^) were attached to the finger-tips of a nitrile rubber glove using Locktite 406 adhesive. For interconnects, conductive silver coated threads were used for each TENG. The threads were finally covered with black tape.

### Material detection using the glove

For this process, 7 different materials (Kapton tape, Cu foil, nitrile, polyamide membrane, polysulfone (PES) membrane, paper and nitrocellulose membrane) were repeatedly tapped with the TENG mounted glove using the index finger. The maximum output voltage and polarity of the signal was recorded by a digital oscilloscope. While tapping, each material was placed on a force plate (FP3, Biometrics Ltd) to record and maintain a constant tapping force of 2 N. Next, a known material (referred to as ‘sample’) was tapped with the same force and the output voltage was recorded. To infer material properties, a simple lookup table algorithm was scripted in MATLAB, to analyse the voltage signals from the sensor. The voltage signals of 7 different materials were stored as .csv files, and were then used as a reference to compare signals from a sample under test (based on amplitude differences and polarity). The experiments were repeated 20 times after which a success rate percent of detection was calculated.

### Electrical measurements

The sheet resistance of the CuNW electrode was measured by a digital multimeter (RSDM 3055). Open circuit voltage (*V*_oc_) of the TENG was measured by a digital oscilloscope (DSO) (MDO3024, Tektronix). The TENG was repeatedly impacted with human finger-tips as well as an Arduino driven pneumatic piston to record the voltage. During finger tapping, the sensor was placed on a force plate (FP4, Biometrics Ltd) which directly recorded the tapping force. The tapping force was varied from 4–30 N with a frequency of 2 Hz and the corresponding *V*_oc_ was measured. The short circuit current (*I*_sc_) was measured under similar conditions using a Keithley 6517B electrometer.

All the participants gave written consent for their involvement during energy harvesting demonstrations using the device.

### Biocompatibility assessment

To assess biocompatibility, cytotoxicity studies were performed using human dermal fibroblast (HDF) cell lines, where the cells were cultured in DMEM media supplemented with FBS (10%) and antibiotic (PSN) (1%) at 37 °C in a humid atmosphere with 5% CO_2_. After 75–80% confluency, the cells were harvested with trypsin (0.025% v/v) and EDTA (0.52 mM in PBS) and were seeded at a desired density to allow them to re-equilibrate for a day before the start of the tests.

Seeded cells (cell density: 10^3^ cells per mL) were then incubated in a sterilised petri-dish with the TENG (cut into a 1 cm^2^ piece) submerged in the media for 72 h. The TENG was sterilised beforehand by washing with absolute ethanol and later UV exposure for 20 min. After incubation, the TENG piece was taken out and the cells were washed with PBS twice and 50 μL live/dead assay stock solution (Calcein AM (2 μM) and Ethidium homodimer (4 μM)) was added to the petri-dish. After 5 min, the cells were again washed with PBS to get rid of the excess dye and thereafter fluorescence images were taken under a fluorescence microscope (EVOS M5000) to evaluate the viability of the cells.

## Author contributions

Biswajoy Bagchi: conceptualization, methodology, validation, investigation, formal analysis, visualization, writing – original draft, Priyankan Datta: methodology, investigation, formal analysis, writing – review and editing, Carmen Salvadores Fernandez: methodology, writing – review and editing, Priya Gupta: writing – review and editing, Shireen Jaufuraully: writing – review and editing, Anna L David: writing – review and editing, Dimitrios Siassakos: writing – review and editing, Adrien Desjardins: writing – review and editing. Manish K Tiwari: conceptualisation, supervision, investigation, project administration, writing – review and editing, resources, funding acquisition.

## Conflicts of interest

The authors declare no conflict of interest.

## Supplementary Material

MH-010-D3MH00404J-s001

MH-010-D3MH00404J-s002

MH-010-D3MH00404J-s003

MH-010-D3MH00404J-s004

MH-010-D3MH00404J-s005

MH-010-D3MH00404J-s006

MH-010-D3MH00404J-s007

MH-010-D3MH00404J-s008

MH-010-D3MH00404J-s009

MH-010-D3MH00404J-s010

MH-010-D3MH00404J-s011

MH-010-D3MH00404J-s012
