# Supplementary material for: Flexible triboelectric nanogenerators using transparent copper nanowire electrodes: energy harvesting, sensing human activities and material recognition
Source: Mater Horiz. 2023 May 15;10(8):3124–34. doi: 10.1039/d3mh00404j (PMC10389064; doi:10.1039/d3mh00404j)
Supplement: MH-010-D3MH00404J-s001 [file MH-010-D3MH00404J-s001.pdf]

Electronic Supplementary Material (ESI) for Materials Horizons.
This journal is © The Royal Society of Chemistry 2023

Electronic Supplementary Information

Flexible triboelectric nanogenerators using transparent copper nanowires electrodes: Energy harvesting, sensing human activities and material recognition

Biswajoy Bagchi[1,2], Priyankan Datta[1,2], Carmen Salvadores Fernandez[1,2], Priya Gupta[1,2], Shireen Jaufuraully[1,3], Anna L. David[1,3,4], Dimitrios Siassakos[1,3,4], Adrien Desjardins[1,5], Manish K Tiwari*[1,2]

[1]Wellcome/EPSRC Centre for Interventional and Surgical Sciences, UCL, London, W1W 7TS, UK

[2]Nanoengineered Systems Laboratory, UCL Mechanical Engineering, London, WC1E 7JE, UK

[3]Elizabeth Garrett Anderson Institute for Women's Health, UCL, London WC1E 6AU

[4]NIHR Biomedical Research Centre at UCL

[5]Department of Medical Physics and Biomedical Engineering, University College London, Gower Street, London, WC1E 6BT, UK

*Corresponding author

E-mail: m.tiwari@ucl.ac.uk, Contact: +442031081056

Comparison with previous Cu and Ag nanostructured electrode based TENGs

Copper and silver based nanostructured electrodes have been implemented in previous TENGs by using various combinations of PDMS and additives such as graphene under both single and double electrode mode. However., a comparative analysis of parameters such as output voltage, current, power density and electrode surface area shows that our copper nanowire based TENG exhibits highest power density obtained from human finger tapping in single electrode mode with an active area of 6 cm^2 (see table S1). In contrast to previously reported methods, our approach utilises oxygen stable copper nanowires (obtained after treatment with lactic acid) without any additive or additional processing steps which benefits from the high charge collecting capacity of the pristine metallic copper. This green approach has inherent advantages of facile synthesis, high conductivity and stability which is reflected in the TENG performance. Finally, although it was not investigated, the rough microstructure of CuNW may generate additional charge at the PDMS-Cu interface during mechanical impact augmenting TENG output.

Table S1: Comparative performance for silver and copper-based TENGs

TENG	V_{oc} (V)	I_{sc} (μA)	Power Density (W/m^2)	Area (cm^2)	Mode of operation

**Ag nanowire-PDMS-TPU**[1]	120	11.6	2.5	4	Double electrode
**Ag nanowire-PDMS**[2]	330	15.5	2.5	16	Single electrode
**Welded Ag nanowire-PDMS**[3]	66	8.5	0.44	16	Single electrode
**Cu nanowire-Cu mesh- PDMS**[4]	155	133	10.3	9	Single electrode
**Sand-paper templated copper-PDMS**[5]	200	1	4.36	9	Double electrode
**Cu nanowire/rGO-PDMS**[6]	125	N. A.	N. A.	4	Double electrode
**Cu nanowire/single crystal graphene-resin**[7]	62	3.6	N.A.	18.75	Double electrode
**Cu nanowire/PDMS**[8]	45	N. A.	0.314	4	Single electrode
**Cu nanowire-Ecoflex (normal)**	200	3.4	10.67	6	Single electrode
**Cu nanowire-Ecoflex (transparent)**<br>**Present work**	125	1.1	3.37	6	

Performance of transparent TENG

The transparent TENG was fabricated simply by using a diluted concentration of CuNW (10 times less than the original TENG). Understandably, the electrical output and performance was low as represented in Figure S1 due the less amount of copper present in the electrode. While the power density may not be high enough for energy harvesting applications, these can be potential candidate for implantable TENG-based sensors as less concentration of copper translates to lower cytotoxicity in biological tissues as observed in our previous study[9].

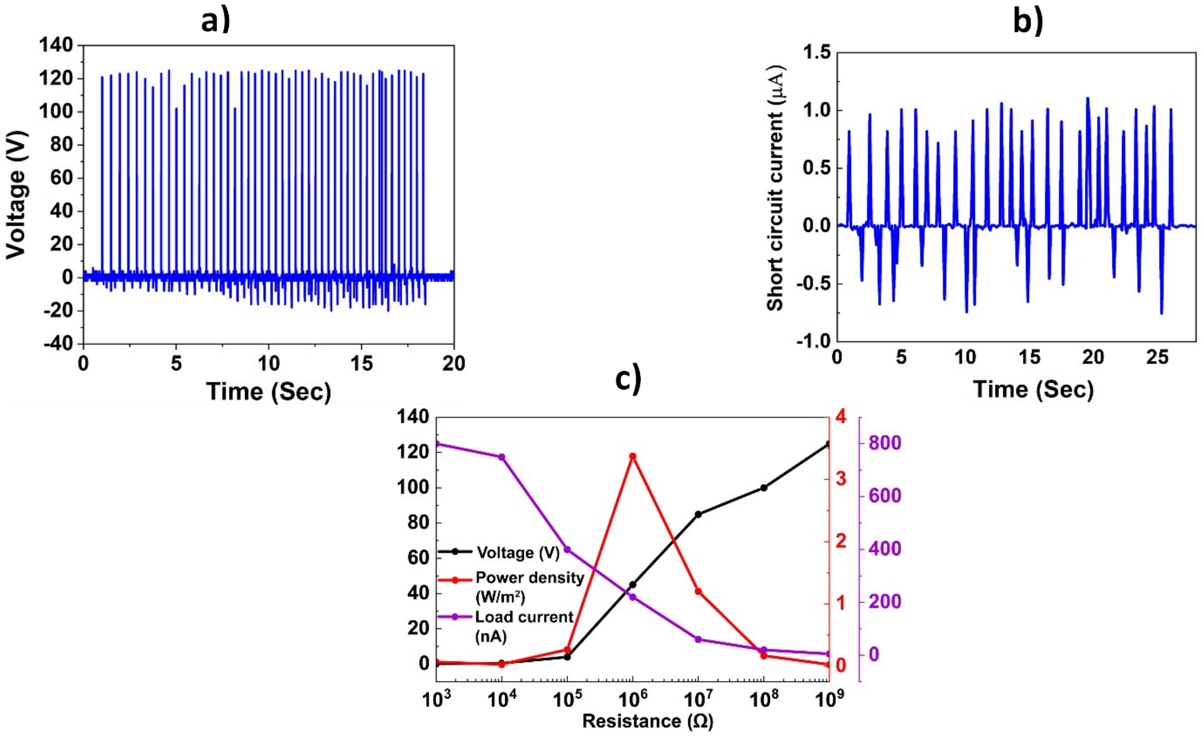

Figure S1: Performance of transparent TENG showing a) open circuit voltage (V_{oc}), b) short circuit current (I_{sc}) and c) variation of instantaneous current (I), voltage (V) and power density (with load resistance) under human finger tapping with a force of 30N.

Substrate versatility of TENG

The TENG can be fabricated using any adhesive flexible substrate which can be tailored for specific applications. For example, different commercial adhesive tapes were used as a substrate, on which CuNW was deposited to make TENGs of variable robustness and transparency. This allows for a wide range of flexible or even stretchable substrates that can used to make conformable TENGs for energy harvesting and sensing from human body movements.

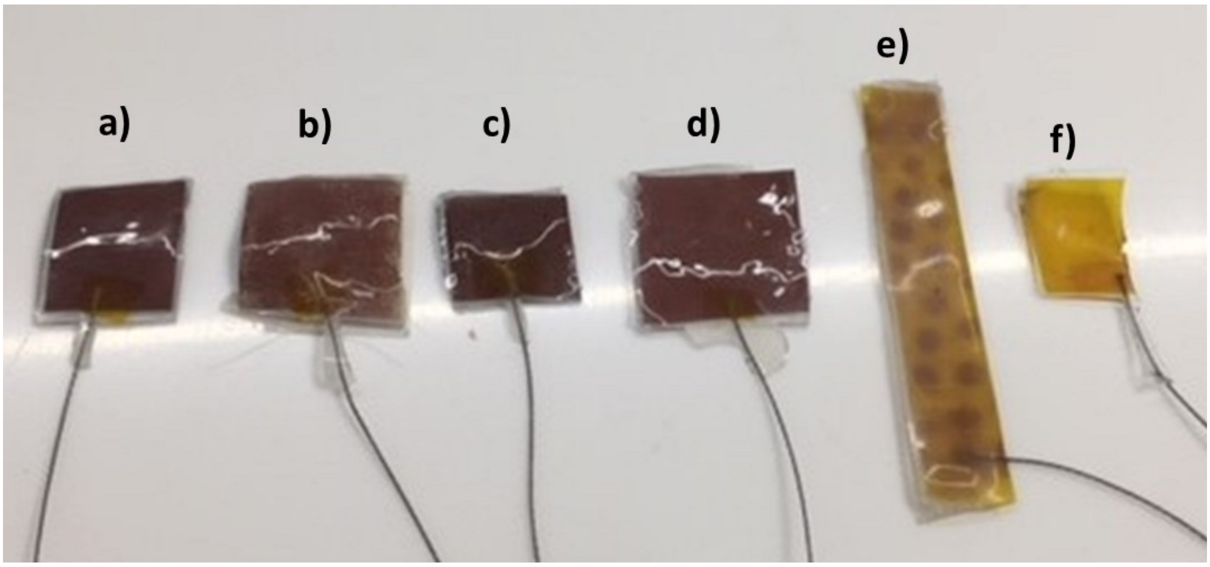

Figure S2: Flexible TENGs fabricated based on different adhesive substrate-a) insulating tape, b) masking tape, c) Gorilla tape, d) double sided tape and e) and f) transparent Kapton tape.

Output from different triboelectric materials

To further highlight the versatility of the TENG, voltage output was measured against some polymeric materials which are conventionally used in packaging, textile and healthcare applications. The TENG was tapped with nylon based textiles, rubber surgical gloves, and PES filter membrane to show the potential of energy harvesting from commonly used materials.

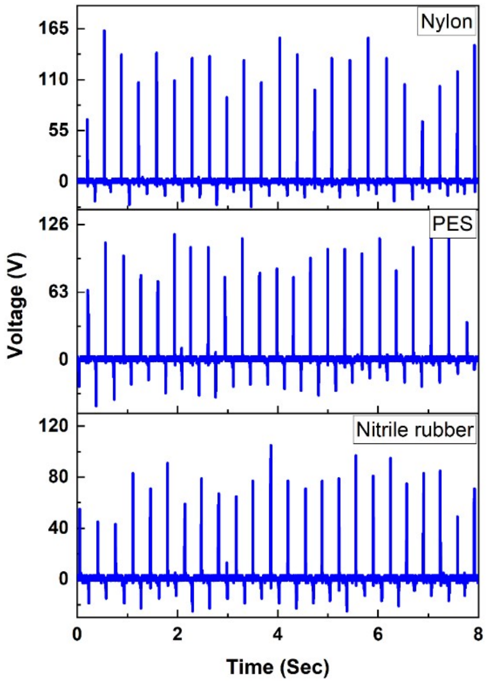

Figure S3: Open circuit voltage (V_{oc}) obtained from TENG upon contact with different surfaces under single electrode mode. PES- Polyether sulphone

Code for Morse code generation using TENG

The following algorithm was used to convert the voltage output from the TENG during tapping with finger to generate and transmit Morse codes on a laptop. Each alphabet was assigned as a ‘dot’ or ‘dash’ (following Morse code) value based on the intensity of the output voltage. The TENG successfully generated voltage outputs consistent with the assigned alphabets to form common words on the laptop screen.

```
#%%
# Find number of peaks in signal
from pathlib import Path
import numpy as np
import pandas as pd
import matplotlib.pyplot as plt
from scipy.signal import find_peaks

Signals = pd.read_csv(Path().joinpath('Morse code test.csv'))

for column in Signals:
    df = Signals[column]
    df = df.dropna()
```

```
    fs = 20
    index = df.index
    number_of_rows = len(index)
    N = number_of_rows
    time = []
    for i in range(N):
        time.append(i/fs)

    peaks1, _ = find_peaks(df)
    feature1 = len(peaks1)

    peaksresized = peaks1.resize((4), refcheck=False)
    peaks1new = np.where(peaks1 == 0, 1, peaks1)

    voltage1 = df[peaks1new[0]]
    voltage2 = df[peaks1new[1]]
    voltage3 = df[peaks1new[2]]
    voltage4 = df[peaks1new[3]]

    if peaks1new[1] == 1:
        feature2 = 1
    else:
        if voltage1 / voltage2 > 1.5:
            feature2 = 2
        else:
            if voltage2 / voltage1 > 1.5:
                feature2 = 3
            else:
                feature2 = 4

    if peaks1new[2] == 1:
        feature3 = 1
    else:
        if voltage2 / voltage3 > 1.5:
            feature3 = 2
        else:
            if voltage3 / voltage2 > 1.5:
                feature3 = 3
            else:
                feature3 = 4

    if peaks1new[3] == 1:
        feature4 = 1
    else:
        if voltage3 / voltage4 > 1.5:
            feature4 = 2
        else:
            if voltage4 / voltage3 > 1.5:
                feature4 = 3
            else:
                feature4 = 4

    if feature1 == 2 and feature2 == 3 and feature3 == 1 and feature4 == 1:
        print("A")
    else:
        if feature1 == 4 and feature2 == 2 and feature3 == 4 and feature4
== 4:
            print("B")
        else:
            if feature1 == 4 and feature2 == 2 and feature3 == 3 and
feature4 == 2:
```

```
                print("C")
            else:
                if feature1 == 3 and feature2 == 2 and feature3 == 4 and 
feature4 == 1:
                    print("D")
                else:
                    if feature1 == 1 and feature2 == 1 and feature3 == 1 
and feature4 == 1:
                        print("E")
                    else:
                        if feature1 == 4 and feature2 == 4 and feature3 == 
3 and feature4 == 2:
                            print("F")
                        else:
                            if feature1 == 3 and feature2 == 4 and feature3 
== 3 and feature4 == 1:
                                print("G")
                            else:
                                if feature1 == 4 and feature2 == 4 and 
feature3 == 4 and feature4 == 4:
                                    print("H")
                                else:
                                    if feature1 == 2 and feature2 == 4 and 
feature3 == 1 and feature4 == 1:
                                        print("I")
                                    else:
                                        if feature1 == 4 and feature2 == 3 
and feature3 == 4 and feature4 == 4:
                                            print("J")
                                        else:
                                            if feature1 == 3 and feature2 
== 2 and feature3 == 3 and feature4 == 1:
                                                print("K")
                                            else:
                                                if feature1 == 4 and 
feature2 == 3 and feature3 == 2 and feature4 == 4:
                                                    print("L")
                                                else:
                                                    if feature1 == 2 and 
feature2 == 4 and feature3 == 1 and feature4 == 1:
                                                        print("M")
                                                    else:
                                                        if feature1 == 2 
and feature2 == 2 and feature3 == 1 and feature4 == 1:
                                                            print("N")
                                                        else:
                                                            if feature1 == 
3 and feature2 == 4 and feature3 == 4 and feature4 == 1:

print("WEISS")
                                                            else:
                                                                if feature1 
== 4 and feature2 == 3 and feature3 == 4 and feature4 == 2:

print("P")
                                                                else:
                                                                    if 
feature1 == 4 and feature2 == 4 and feature3 == 3 and feature4 == 4:

print("Q")
                                                                    else:
```

```
                                                                   if
feature1 == 3 and feature2 == 3 and feature3 == 2 and feature4 == 1:
print("R")
else:
if feature1 == 3 and feature2 == 4 and feature3 == 4 and feature4 == 1:
print("S")
else:
if feature1 == 1 and feature2 == 1 and feature3 == 1 and feature4 == 1:
print("T")
else:
if feature1 == 3 and feature2 == 4 and feature3 == 3 and feature4 == 1:
print("U")
else:
if feature1 == 4 and feature2 == 4 and feature3 == 4 and feature4 == 3:
print("V")
else:
if feature1 == 3 and feature2 == 3 and feature3 == 4 and feature4 == 1:
print("W")
else:
if feature1 == 4 and feature2 == 2 and feature3 == 4 and feature4 == 3:
print("X")
else:
if feature1 == 4 and feature2 == 2 and feature3 == 3 and feature4 == 4:
print("Y")
else:
print("Z")
```

Figure S4: Script for generating 'words' during tapping on TENG using Python language in PyCharm.

Voltage pattern from holding different objects

The sensor mounted glove was used to hold and interact with different objects which produced voltage patterns and peak intensities based on respective triboelectric pairs (metal-Ecoflex, plastic-Ecoflex, glass-Ecoflex). Detailed analysis of these voltage outputs (frequency, intensity and overall pattern) may be beneficial to extract information about different human-device interactions and potentially aid in object recognition and robotic surgery in future.

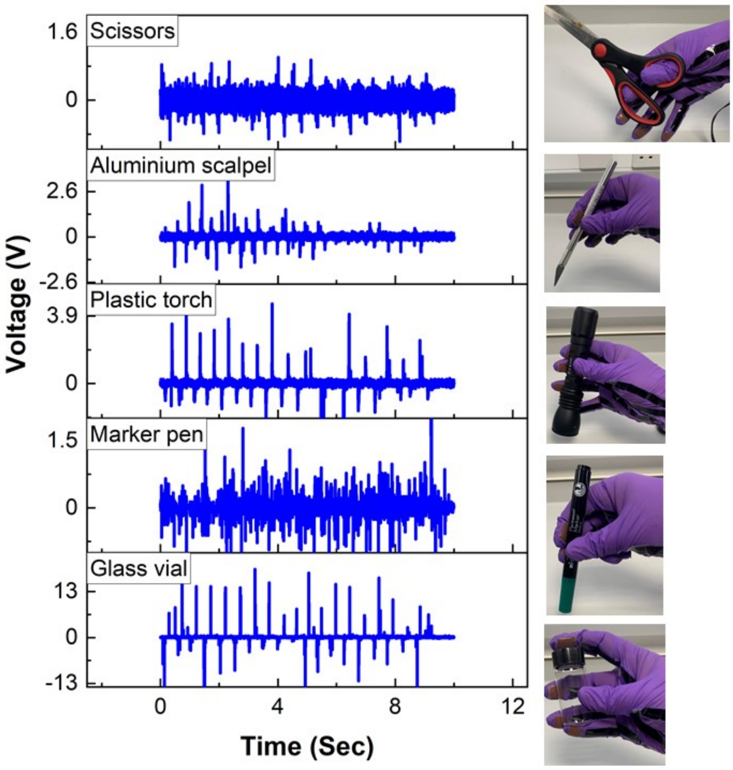

Figure S5: Open circuit voltage (V_{oc}) obtained from TENG sensorized glove upon grasping different objects in single electrode mode.

Force calibration of TENG

Force was calibrated by applying a known impact force on the TENG surface and then simultaneously recording the voltage output (by digital oscilloscope and force (by using a force plate- see section 2.6). The impact force was applied 20 times and varied from 3-30 N. Figure S6 shows the plot of Voltage output vs applied force. The output voltage acquired from the TENG increased linearly with force showing a working range of 5-30 N.

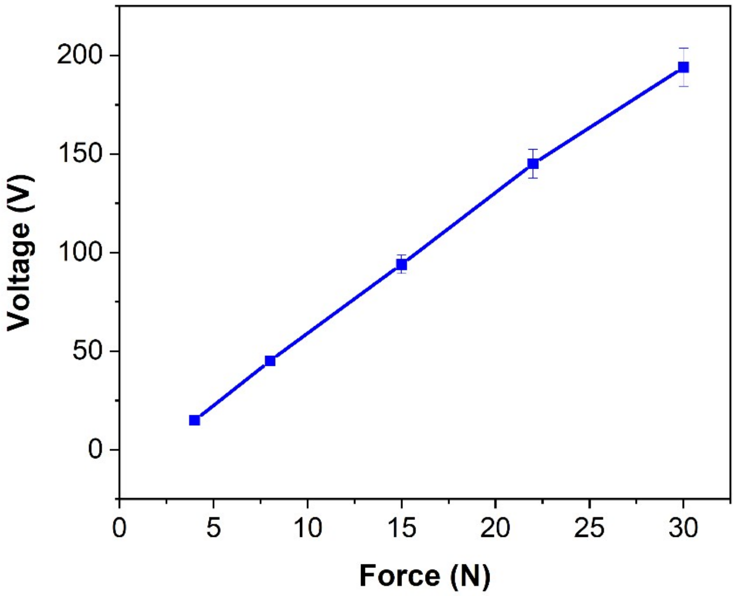

Figure S6: Voltage (V_{oc}) vs Force calibration curve using TENG. The TENG was tapped with different controlled forces (force was measured directly by placing the device on a force plate) and the corresponding output voltage was recorded by an oscilloscope.

Material identification with TENG mounted glove

An attempt has been made to use TENG sensor mounted glove as a simple analytical tool to identify material characteristics by exploiting differential triboelectric charge generation between Ecoflex and some commonly used known materials. The voltage output obtained after tapping on each material is then analysed based on two identifying parameters like 'polarity' and 'intensity' (see section 3) and finally compared to an unknown material. Material is then identified based on minimal difference between the above mentioned parameters. The material polyimide was successfully (98% success rate) identified by comparing with 7 different materials based on output voltage and polarity. Although this is a crude and qualitative method, testing with wider range of materials followed by careful analysis of the voltage/current outputs using stringent conditions and comparing them in the triboelectric series may open up possibilities to develop touch based material identification strategies in future[10].

Table S2: Material identification by processing touch based signals using flexible TENG mounted glove.

	Sample	Kapton tape	Cu foil	Nitrile glove	Polyamide (Nylon)	PES	Paper	Nitrocellulose
**Maximal value (V)**	**3.7**	**3.5**	**6**	**9.8**	**8.2**	**4.5**	**8.5**	**8**
**Sample polarity**	+	+	+	+	+	+	+	
**Difference between sample and material**		**0.2**	**2.3**	**6.1**	**4.5**	**0.8**	**4.6**	**4.3**
**Success Rate %**	**98**<br>**Material detected – Polyimide filter membrane**							

Video S1: V_{oc} obtained from TENG under repeated impact (Force- 30N, frequency- 4 Hz) from a pneumatically driven plunger fitted with PES membrane in single-electrode mode. The TENG is tested for 8000 cycles showing stable voltage output.

Video S2: Robustness and flexibility testing by stretching and bending.

Video S3: Powering up 115 LEDs by finger tapping on flexible TENG.

Video S4: Powering up 50 LEDs by finger tapping on transparent TENG.

Video S5: Powering up LED screen of a digital calculator by TENG.

Video S6: Powering up LED screen of a digital thermometer by TENG.

Video S7: Morse code generation by TENG showing coding and sending short text messages after processing through PyCharm.

Video S8: TENG detecting stretching of skin.

Video S9: TENG detecting bending of wrist.

Video S10: TENG detecting finger joint movement.

Video S11: TENG based sensorised surgical glove showing variation of voltage output from tapping on different surfaces.

References

1 H. Niu, X. Du, S. Zhao, Z. Yuan, X. Zhang, R. Cao, Y. Yin, C. Zhang, T. Zhouab, C. Li, *RSC Adv.*, 2018, **8**, 30661.

2 L. Cheng, Y. Xi, C. Hu, X. Yue, G. Wang, *EHS*, 2016, **3**, 91–99.

3 X. Liang, T. Zhao, W. Jiang, X. Yu, Y. Hu, P. Zhu, H. Zheng, R. Sun, C. Wong, *Nano Energy*, 2019, **59**, 508.

4 Q. Zhou, J. Kim, K. Han, S. Oh, S. Umrao, E. J. Chae, Il. Oh, *Nano Energy*, 2019, **59**, 120.

5 X. Zhang, G. Li, G. Wang, J. Tian, Y. Liu, D. Ye, Z. Liu, H. Zhang, J. Han, *ACS Sustainable Chem*. Eng., 2018, **6**, 2283.

6 G. Li, G. Wang, Y. Cai, N. Sun, F. Li, H. Zhou, H. Zhao, X. Zhang, J. Han, Y. Yang, *Nano Energy*, 2020, **75**, 104918.

7 J. Wang, Z. Zhang, S. Wang, R. Zhang, Y. Guo, G. Cheng, Y. Gu, K. Liu, K. Chen, *Nano Energy*, 2020, **71**, 104638.

8 G. Li, Y. Cai, G. Wang, N. Sun, F. Li, H. Zhou, X. Zhang, H. Zhao, Y. Wang, J. Han, Y. Yang, *Nano Energy*, 2022, **99**, 107429.

9 B. Bagchi, C. Fernandez, M. Bhatti, L. Ciric, L. Lovat, M. K. Tiwari, *J. Colloid Interface Sci*. 2020, **168**, 30.

10 Y. Wang, H. Wu, L. Xu, H. Zhang, Y. Yang, Z. L. Wang, *Sci. Adv.* 2020, **6**, 9083.
